# Artificial Base-Directed In Vivo Formulation of Aptamer–Drug Conjugates with Albumin for Long Circulation and Targeted Delivery

**DOI:** 10.3390/pharmaceutics14122781

**Published:** 2022-12-13

**Authors:** Yang Sun, Xinyao Geng, Yue Ma, Yu Qin, Shangjiu Hu, Yuquan Xie, Ruowen Wang

**Affiliations:** 1 Institute of Molecular Medicine (IMM), Renji Hospital, State Key Laboratory of Oncogenes and Related Genes, School of Medicine, Shanghai Jiao Tong University, Shanghai 200127, China; 2 Department of Cardiology, Renji Hospital, State Key Laboratory of Oncogenes and Related Genes, School of Medicine, Shanghai Jiao Tong University, Shanghai 200127, China

**Keywords:** aptamer–drug conjugates, albumin complex, cancer therapy, targeted delivery

## Abstract

Aptamer–drug conjugates (ApDCs) are potential targeted pharmaceutics, but their clinical applications are hampered by fast clearance in blood. Herein we report the construction of ApDCs modified with artificial base F and the study of biological activities. Two types of F-base-modified ApDCs were prepared, Sgc8-paclitaxel by conjugation and Sgc8-gemcitabine, by automated solid-phase synthesis. In vitro experiments showed that F-base-modified ApDCs retain the specificity of the aptamer to target cells and the biological stability is improved. In vivo studies demonstrated that the circulatory time is increased by up to 55 h or longer, as the incorporated F base leads to a stable ApDC-albumin complex as the formulation for targeted delivery. Moreover, conjugated drug molecules were released efficiently and the drug (paclitaxel) concentration in the tumor site was improved. The results demonstrate that an F-base-directed ApDC-albumin complex is a potential platform for drug delivery and targeted cancer therapy.

## 1. Introduction

Chemotherapy is one of the major types of cancer treatments that uses anticancer drugs [1]. Due to severe side effects caused by chemotherapeutics, targeted drug delivery has been extensively explored during the last decades [2]. The conjugates of drugs with targeted ligands can enhance the efficacy and decrease the nonspecific toxicity of drugs, as illustrated by several approved antibody–drug conjugates (ADCs) [3]. Aptamers are single-stranded oligonucleotides selected through SELEX technology, which can specifically recognize and bind to their targets with high affinity [4,5,6]. It has been demonstrated that aptamer–drug conjugates (ApDCs) are a potential targeted drug delivery system for cancer therapy, which are constructed through a chemical approach [7,8,9,10,11,12]. 

Paclitaxel (PTX) is a potent chemotherapeutic that effectively prevents mitosis and thus inhibits the growth of cancer cells by binding to and stabilizing microtubules [13,14]. However, its clinical applications are limited by the serious side effects and poor aqueous solubility [15,16]. Albumin-bound paclitaxel (Nab-PTX) has disposed of the solubility issue, showing an obvious advantage to different indications [17]. PTX has been widely used as a cytotoxic payload in the construction of targeted delivery systems, such as conjugates with ligand molecules (antibodies, peptides, aptamers) and complexes with nanoparticles [18,19,20,21]. Zhang et al. reported the conjugate of PTX with aptamer AS1411 via an enzymatical cleavage linker and demonstrated the potential application of the targeted drug delivery system for pancreatic cancer [22]. 

Compared with antibodies, aptamers are superior in terms of their immunogenicity, manufacture, and chemical modification [5,6]. However, the clinical applications of aptamer therapeutics have been affected by their short in vivo half-life and their fast enzymatic degradation and renal excretion [23]. Efforts have been devoted to enhancing the in vivo stability of aptamers. Chemical modification is a general approach to improving the biostability of aptamers to meet clinical requirements [24,25]. For example, Macugen, as the first aptamer therapeutic, is modified with polyethylene glycol moiety to increase in vivo stability [26]. Many delivery systems have also been developed to protect aptamer therapeutics from fast enzymatic degradation and renal excretion. Recently, we developed a general approach to regulate the interaction of oligonucleotides with albumins by incorporating an F base. The incorporation of two hydrophobic F bases at both the 3′-end and 5′-end of an oligonucleotide leads to the formation of a stable complex with albumin, conferring the oligonucleotide longer circulatory time than classical C18 and cholesterol modifications [27].

**Scheme 1:**
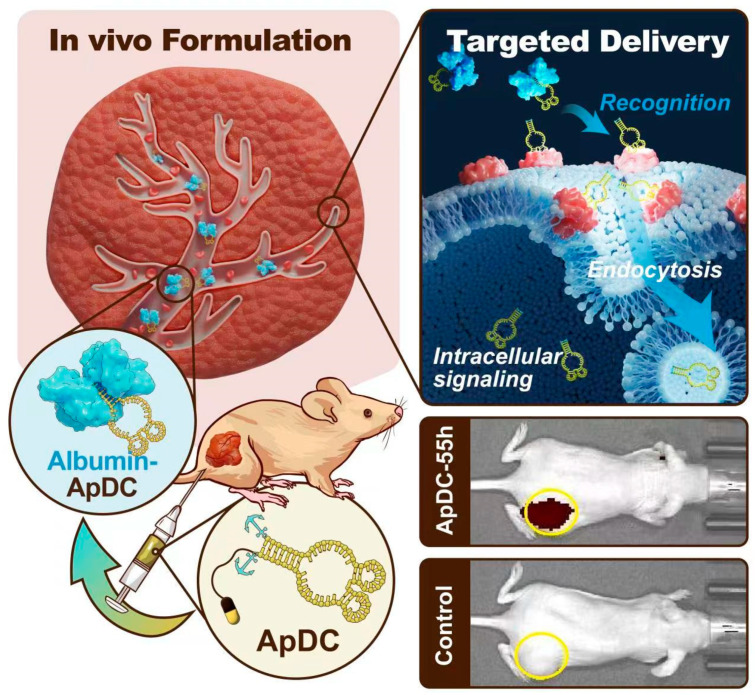
Schematic illustration of the targeted delivery system of artificial base-modified ApDC. After intravenous injection of ApDC, terminal F bases as anchors would direct the formulation of the albumin-ApDC complex within the blood circulation; ApDC could be sheltered from fast enzymatic degradation and renal excretion, and accumulate in the tumor site.

We hypothesize that the introduction of F bases at both ends of an aptamer–drug conjugate (ApDC) might elongate the in vivo half-life and circulatory time. When the ApDC is injected into the circulatory system, F bases will anchor to serum albumin (Scheme 1) generating a stable albumin-ApDC complex (AAC). The in vivo formulation could be an ideal targeted delivery system, as the AAC can shelter the ApDC from fast enzymatic degradation and renal excretion. The complex may accumulate at the tumor site through the EPR (enhanced permeability and retention) effect [28]. Then the ApDC is further located on the tumor cell surface and endocytosed via the specific recognizing and binding interaction of the aptamer with its antigen, which is generally a membrane protein overexpressed in tumor cells [29,30]. Drug molecules are released from the ApDC inside cells, inhibiting cell proliferation. In this way, the F-modified ApDC could be efficiently delivered to the target with enhanced efficacy for its elongated circulatory time. Compared with classical delivery systems such as lipid nanoparticles, F-base-directed in vivo formulation is a much simpler approach. Therefore, the modification with the F base may present a novel platform for the targeted delivery of oligonucleotide-based therapeutics with elongated circulation. 

## 2. Materials and Methods

### 2.1. General Information

Unless otherwise noted below, all commercially available reagents and solvents were purchased from Tansoole (Shanghai, China) and used without further purification. NMR spectra were recorded on a Bruker AM400 spectrometer. All oligonucleotides were synthesized and purified by Sangon Biotech (Shanghai, China) or Biosyn Biotech (Suzhou, China), and the sequences are listed in Table S1. Nab-PTX was purchased from CSPC Pharmaceutical Group Limited. Bovine serum albumin (BSA) and Cell counting kit 8 (CCK-8) were purchased from Beyotime Biotech. All aptamer–drug conjugates were purified by reversed-phase HPLC with a C18 column using 0.1 M triethylamine acetate (TEAA) and acetonitrile as eluent.

### 2.2. Cell Culture 

All cells were cultured at 37 °C with 5% CO_2_ and 95% humidity. HCT116 cells were maintained in RPMI-1640 medium with 10% FBS (Gibco). MIA PaCa-2 cells were maintained in a DMEM medium supplemented with 10% FBS. HK-2 cells were maintained in MEM medium with 10% FBS. All cell lines were preserved in our laboratory.

### 2.3. PTX-Linker Synthesis 

In the presence of 2, 6-dimethylpyridine (28.2 mg, 0.263 mmol), 3-Maleimidopropionic acid (23.4 mg, 0.125 mmol) was added dropwise to a solution of paclitaxel (106 mg, 0.125 mmol) in CH_2_Cl_2_ (5 mL) at 0 °C. The mixture was stirred at RT for 18 h. When the crude material was consumed, as shown by TLC, the reaction was diluted with CH_2_Cl_2_ (20 mL) and washed with saturated sodium bicarbonate (10 mL) and saturated saline (10 mL). After drying over anhydrous sodium sulfate, the solution was concentrated by a rotavapor, and the residue was dissolved in CH_2_Cl_2_ (1 mLX2) and loaded to a flash column. The purification gave PTX precursor as a white powder (75.4 mg, 60% yield).

### 2.4. Aptamer-PTX Conjugate Synthesis 

PTX precursor solution (1200 μL, 2 mM in DMF) was added into thiol-modified DNA (customized from Suzhou Biosyn) solution (240 nmol in 1200 μL double distilled H_2_O, 200 μM). After ultrasonic vibration (Kunshan Ultrasonic instrument (Kunshan, China), KQ-100KDB) for one hour, the mixture was then gently stirred at room temperature for 24 h. The DNA products were precipitated with NaCl (3 M, 132 µL) and ethanol (6000 µL). The DNA precipitation was collected by removing the supernatant and then dissolved in 1400 µL ddH_2_O and purified by HPLC with 0.1 M TEAA and acetonitrile as the eluent at the detection wavelength of 260 nm. The reaction product was finally identified by mass spectrometry (Sangon Biotech).

### 2.5. Flow Cytometric Analysis

The binding affinity of ApDCs was studied by flow cytometry (BD FACSVerse, San Jose, CA, USA). In total, 1 × 10^6^ cells were seeded in a T75 culture bottle for 24 h of culturing, then were treated with 500 μL 0.2% EDTA. After three times washing with 1 mL washing buffer, cells (3 × 10^5^) were incubated with 250 nM cy5-labeled aptamer derivatives in 200 μL binding buffer (50 pmol) on ice for 30 min. Finally, the cells were resuspended in 200 μL washing buffer for flow cytometric analysis. Library (Lib) was used as a negative control, and Sgc8 was used as a positive control. The data were analyzed with FlowJo software. 

Washing buffer: 5 mM MgCl_2_ and 4.5 g/L glucose in Dulbecco’s PBS. 

Binding buffer: 5 mM MgCl_2_, 4.5 g/L glucose, 0.1 mg/mL yeast tRNA, and 1 mg/mL BSA in Dulbecco’s PBS.

### 2.6. Confocal Microscopy Imaging

HCT116 and MIA PaCa-2 cells were seeded in glass bottom dishes (NEST Biotechnology, Wuxi, China), respectively, at a density of 1 × 10^5^ cells per well and allowed to grow overnight. A total of 500 nM cy5-labeled Sgc8-F-PTX or cy5-labeled Lib was incubated with cells in 500 μL binding buffer at 4 °C for 0.5 h. Then, the buffer was removed, and cells were washed three times with washing buffer (1 mL/time) and replaced with 1 mL culture medium supplemented with 10% FBS. After incubating at 37 °C for 1 h, the fluorescence of the sample was visualized by confocal microscopy (Leica TCS SP8, ex: 650 nm, em: 670 nm).

### 2.7. Cell Viability Assay

HCT116 and MIA PaCa-2 cells were seeded at a density of 1 × 10^4^ cells in 96-well plates (Corning) and allowed to grow for 24 h. Cells were incubated with different concentrations (1, 2, 5, 10, 20, 50, 100, 200 nM) of drugs that were prepared in a culture medium for 72 h at 37 °C. Cell viability was tested using a CCK-8 kit by monitoring the absorbance at 450 nm with a microplate reader (Biotek, Synergy H1). Finally, IC_50_ values of drugs were calculated using GraphPad based on the viability curve data. Data were mean ± SD, *n* = 3.

### 2.8. Serum Stability Analysis 

To investigate the serum stability of aptamers, 1 μM cy5-labeled Sgc8 and Sgc8-F-PTX were incubated with DMEM supplemented with 10% fetal bovine serum (FBS) at 37 °C at different times. Then, samples were heated at 95 °C for 5 min to stop the reaction and subsequently stored at −80 °C. Next, samples were analyzed with 10% polyacrylamide gel electrophoresis (PAGE). Finally, the gels were imaged using the GE Amersham Imager 680R.

### 2.9. In Vivo and Ex Vivo Imaging

BALB/c nude mice (4–6 weeks) were provided by the Jihui laboratory animal center (Shanghai, China). All mice were well housed and fed under specific pathogen-free (SPF) conditions. The procedures of all in vivo studies gained ethical approval from the Institutional Animal Care and Use Committee of Shanghai Jiao Tong University School of Medicine. Mice were euthanized when the tumor size reached more than 2500 mm^3^.

In total, 4 × 10^6^ MIA PaCa-2 cells were injected subcutaneously into the right back of five-week-old female BALB/c nude mice. When the volumes of tumors reached 300–400 mm^3^, mice were randomly divided into two groups and intravenously injected with 1 nmol (100 μL of 10 μM) cy5-labeled Sgc8 and Sgc8-F-PTX, respectively. Then, the nude mice were anesthetized and imaged with the IVIS Lumina XR imaging system (ex: 640 nm; filter: 670 nm) at predetermined time points including 0, 0.5, 1, 2, 3, 4, 6, 10, 24, 48, and 55 h. The mice were euthanatized 55 h after injection. Tumors and major organs (hearts, lungs, livers, spleens, and kidneys) were collected for imaging. Fluorescence intensity was analyzed with GraphPad.

### 2.10. Tumor PTX Concentration Analysis 

When the volume of tumors reached about 300 mm^3^, mice were randomly divided into four groups. Sgc8-F-PTX, Nab-PTX, and Sgc8-F, at a dosage of 2.34 μmol/kg (with the equivalent PTX concentration of 2 mg/kg), were administered individually to each mice group by intravenous tail injection once a week for 2 weeks, DPBS was used as control. After 2 weeks of therapy, the DNA dose was increased to 4.68 μmol/kg (equal to a dose of 4 mg/kg for PTX) during the next 2 weeks. After administration, the mice were observed for another 2 weeks. Tumor size and body weight were monitored twice a week. 

After 6 weeks of therapy, the tumors of all treatment groups were collected and weighed. The concentration of PTX in tumors was studied by Sangon Biotech with UPLC-ESI-MS/MS. Drug content was analyzed with GraphPad.

## 3. Results and Discussion 

### 3.1. Design and Synthesis of Aptamer–Paclitaxel Conjugates

**Figure 1:**
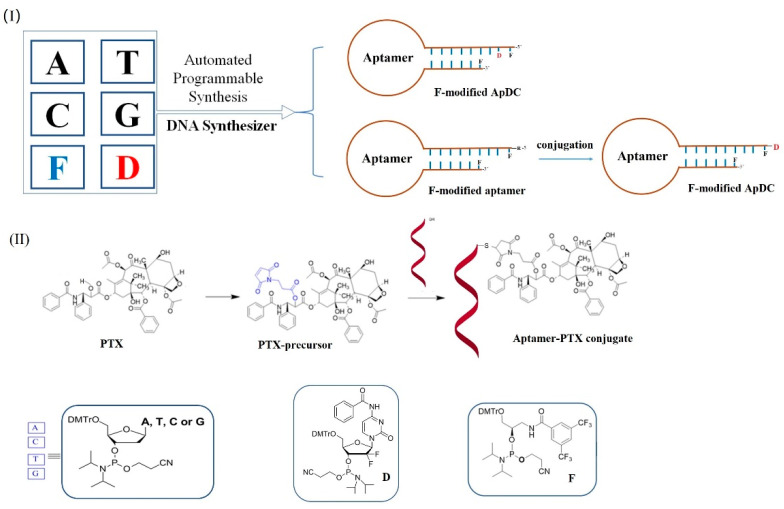
(**I**) Schematic illustration of automated aptamer–drug conjugate synthesis. (**II**) The synthesis of PTX precursor and the synthesis of ApDC by conjugation of the thiol-modified aptamer.

As reported recently, the modification of oligonucleotides (ONs) with hydrophobic F bases at both terminals confers ONs’ improved nuclease resistance ability and elongated circulatory time [27]. As shown in Figure 1, F-modified ApDCs can be prepared by both automated programmable synthesis and traditional conjugation. Using solid-phase reagents containing F base (phosphoramidite F), the reagent containing drug moieties (D phosphoramidite), and together with ATCG phosphoramidites, F-modified ApDCs may be prepared automatedly and efficiently by solid-phase synthesis. On the other side, it is impossible to incorporate some small molecular drugs into ONs by automated synthesis due to the present technological challenges, and those drugs may be conjugated to aptamers by chemical synthesis in solution. It is not clear yet if the introduction of F bases would confer these ApDCs elongated circulatory time. Moreover, the applications of F-modified ApDCs must find out whether the drug could be released to interact with target cells. Herein, we designed and synthesized a series of ONs (Table S1) to investigate the biological activities. Phosphoramidite D (Figure 1) is the commercially available solid-phase reagent of the drug gemcitabine (D), from which D as a drug unit can be incorporated at any position of ONs. F-modified aptamer–paclitaxel conjugate is prepared from the conjugation of PTX precursor with F-modified aptamer (Figure 1(II)).

PTX precursor was prepared by the reaction of 3-Maleimidopropionic acid with PTX with a yield of 60% as a white powder. The structure was identified by ^1^H-NMR, ^13^C-NMR spectrum, and mass analysis. Thiol-modified aptamer Sgc8 and F-modified Sgc8 derivatives were prepared to construct aptamer–paclitaxel conjugates. The maleimide–thiol chemistry was performed highly efficiently giving a series of ApDCs as designed. The ApDCs were purified by HPLC and identified by mass analysis.

### 3.2. ApDC Internalization Efficiency and Stability

**Figure 2:**
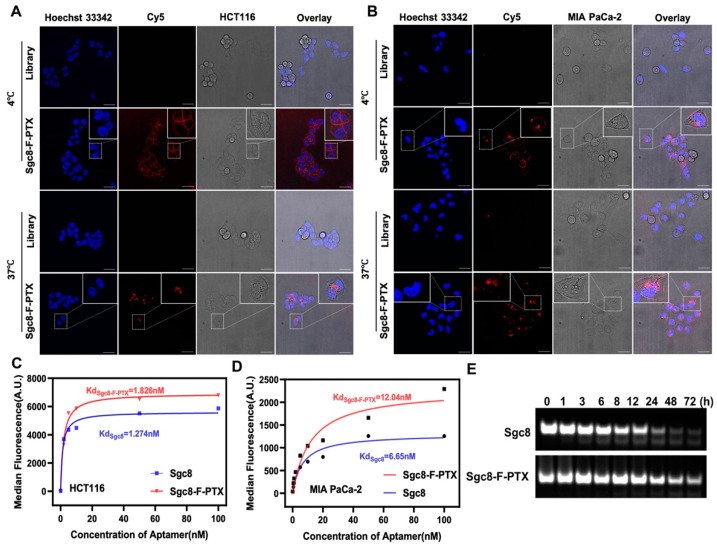
(**A**,**B**) Confocal laser scanning microscopy (CLSM) imaging of cells. HCT116 cells (**A**) and MIA PaCa-2 cells (**B**) were incubated with 500 nM cy5-labeled Sgc8-F-PTX or library at 4 °C for 30 min or at 37 °C for 1 h. The nuclei were stained with Hoechst 33342, the scale bar represents 30 μm. (**C**,**D**) Flow cytometry tests the binding affinity of aptamers. HCT116 cells (**C**) and MIA PaCa-2 cells (**D**) were incubated with 250 nM cy5-modified aptamer derivatives in a binding buffer for 30 min at 4 °C, then the fluorescence of cells was analyzed with flow cytometry. (**E**) PAGE gel analysis of the stability of Sgc8 and Sgc8-F-PTX. Aptamers were incubated with 10% FBS for different times and then analyzed with PAGE.

Internalization efficiency was important for drug delivery, so we first explored the internalization ability of ApDCs by confocal microscopy. As demonstrated in Figure 2A,B, the fluorescence of the cy5-labeled ApDC was attached to the surface of HCT116 and MIA PaCa-2 cells when incubated at 4 °C, suggesting that the ApDC is recognized and bound to the target cancer cell membrane. After 1 h of incubation at 37 °C, the cy5-labeled Sgc8-F-PTX molecules were mainly located inside target cells, implying that they were internalized. In contrast, the library sequence (Lib) showed almost no binding or internalization. Results indicated that Sgc8-F-PTX may trigger internalization in a specific way, making it much more suitable as a candidate for therapeutic applications. To explore whether the ApDC retains the binding affinity of Sgc8, we further analyzed the equilibrium dissociation constant (Kd) of the ApDC by flow cytometry in two cell lines (Figure 2C,D). The results indicated that Sgc8-F-PTX exhibits a similar binding affinity with HCT116 cells (Kd = 1.826 nM) and MIA PaCa-2 cells (12.04 nM) as Sgc8 (HCT116, 1.274 nM and MIA PaCa-2, 6.65 nM). Since stability is a key factor for nucleic acid drugs, we then examined the stability of the ApDC with PAGE. As implied in Figure 2E, Sgc8 gradually disappeared after incubation with 10% FBS for 8 h, while Sgc8-F-PTX remained nearly intact after incubation for 24 h within the same condition. The serum stability results suggested that Sgc8-F-PTX is more stable than natural Sgc8, which is consistent with our previous studies.

### 3.3. Targeting Effect of ApDC

**Figure 3:**
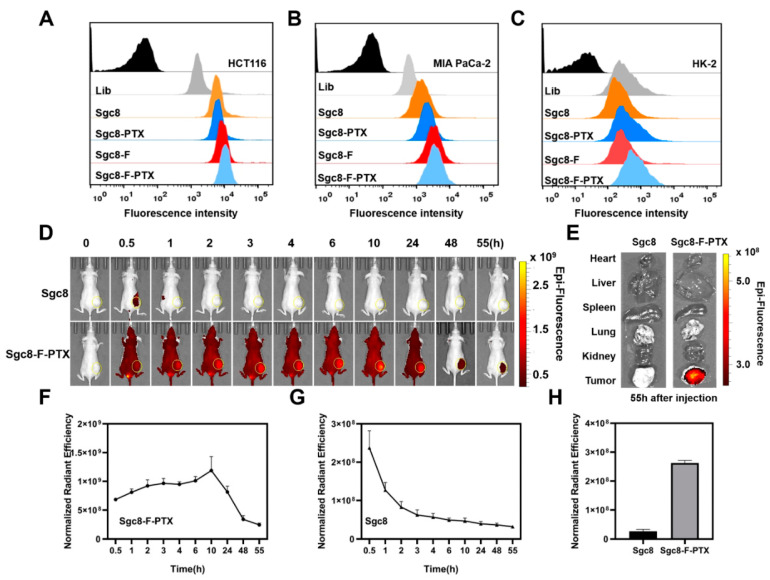
(**A**–**C**) Targeting effect of ApDC on the target cells (**A**, HCT116, and **B**, MIA PaCa-2) and control cells (**C**, HK-2) was analyzed with flow cytometry. Cells were incubated with 250 nM cy5-labeled oligonucleotides at 4 °C for 30 min. (**D**) In vivo fluorescence imaging of MIA PaCa-2 tumor-bearing mice after cy5-labeled Sgc8 or Sgc8-F-PTX were injected intravenously. The tumor sites are marked with yellow circles. (**E**) Ex vivo imaging of the main organs including heart, liver, spleen, lung, and kidney at 55 h post-injection. (**F**) The in vivo average radiant efficiency in tumor sites at different time points after cy5-labeled Sgc8 was injected intravenously, *n* = 3. (**G**) The in vivo average radiant efficiency in tumor sites at different time points after cy5-labeled Sgc8-F-PTX was injected intravenously, *n* = 3. (**H**) The quantitative result of the ex vivo fluorescence intensity in tumor site, *n* = 3.

We hypothesized that the F-modified ApDC would retain the specific targeting property as aptamer Sgc8 and accumulate in the tumor tissue through the albumin-ApDC complex with elongated half-life. To verify the hypothesis, cy5-labeled oligonucleotides were, respectively, incubated with target cells (HCT116 and MIA PaCa-2) and nonspecific cells (HK-2). As shown in Figure 3A,B, the fluorescence of target cells incubated with aptamer and ApDCs (Sgc8-PTX, Sgc8-F-PTX, Sgc8-FG-PTX) showed nearly no difference when analyzed by flow cytometry (Figure S1), while there was no obvious binding with nontarget HK-2 cells (Figure 3C). The results suggested that both the unmodified and F-modified ApDC retained the specific targeting effect of aptamer Sgc8. To further investigate the in vivo targeting effect of ApDCs, cy5-labeled Sgc8 or the F-modified ApDC were injected into the MIA PaCa-2-bearing xenografted mice model through the tail vein, respectively. As shown in Figure 3D,F, Sgc8 accumulated at the tumor site within 0.5 h post-injection and went through a quick renal clearance at about 1 h post-injection, which was consistent with previous studies. In comparison, the fluorescence signal of F-modified ApDC was distributed throughout the whole body at the beginning, and gradually accumulated at the tumor site with an enhanced fluorescence signal at 10 h post-injection and remained robust till 55 h post-injection. Similar phenomena were also observed for the F-modified ApDC incorporated with gemcitabine (Figure S2A,B). At 55 h after intravenous injection, the mice were euthanized and their major organs (heart, liver, spleen, lung, kidney) were collected. It was shown that the fluorescence intensity in tumor tissue collected from the ApDC group was higher than that from the Sgc8 group (Figure 3E,H), which was consistent with the in vivo imaging results. Therefore, it could be concluded that the F-modified ApDCs have a similar targeting effect as the F-modified aptamer Sgc8-F, rendering them as potential targeted drug delivery systems for cancer therapy.

### 3.4. The Inhibition of Cell Proliferation and In Vivo Drug Release

**Figure 4:**
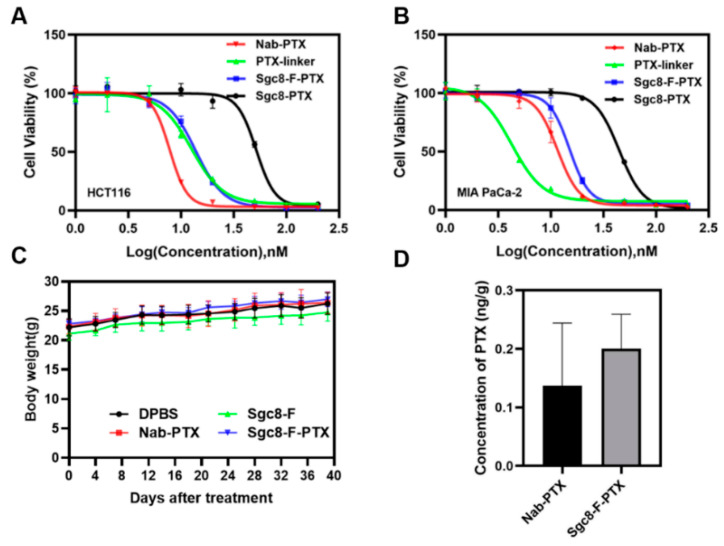
(**A**) Proliferation–inhibitory effects against HCT116 cells and (**B**) MIA PaCa-2 cells; cells were incubated with drugs at different concentrations for 72 h and analyzed with CCK-8 assay, and data were presented as the means ± standard deviation, *n* = 3. (**C**) Body weight of MIA PaCa-2 tumor-bearing mice with different treatments. (**D**) PTX concentration analysis of Nab-PTX- or Sgc8-F-PTX-treated mice; PTX was extracted from the tumor tissue and analyzed with UPLC-ESI-MS/MS.

Since the F-modified ApDC retains the targeting effect of aptamer Sgc8, we further investigated their cytotoxicity and in vivo drug release properties. HCT116 and MIA PaCa-2 cells were incubated with Nab-PTX, PTX–linker, Sgc8-PTX, and Sgc8-F-PTX, respectively, for 48 h. As demonstrated in Figure 4A,B, PTX precursor and Sgc8-F-PTX exhibited good cytotoxicity comparable to Nab-PTX with IC_50_ values around 10 nM (Tables S2 and S3). Sgc8-PTX, the ApDC without F modification, showed an inferior inhibitory effect on cell proliferation with an IC_50_ value of 51.81 nM for HCT116 and 44.72 nM for MIA PaCa-2 cells. The F-modified ApDC incorporated with gemcitabine (Sgc8-FG-PTX) exhibited similar inhibitory activity as gemcitabine (Figure S3). The cytotoxicity experiments indicate that the modification of the F base would not prevent drug molecules from inhibiting cell proliferation, no matter whether the drug is conjugated to the terminal of an aptamer or incorporated into the middle of the oligonucleotide.

To determine the in vivo drug release property of Sgc8-F-PTX, a MIA PaCa-2-bearing mice model was built. Mice with a tumor of about 300 mm^3^ volume were randomly divided into four groups for the administration of DPBS, Sgc8-F-PTX, Nab-PTX, and Sgc8-F, respectively. The drugs were administered at a 2 mg/kg dosage intravenously once a week for 2 weeks, and 4 mg/kg for the next 2 weeks. After administration, the mice were observed for another 2 weeks. As demonstrated in Figure 4C, Sgc8-F-PTX performed good biosecurity. Tumor tissues of the Sgc8-F-PTX group and the Nab-PTX group were collected and PTX concentration was analyzed with UPLC-ESI-MS/MS. Results showed that the concentration of the Sgc8-F-PTX group is higher than that of the Nab-PTX group (Figure 4D), indicating that Sgc8-F-PTX could be a better drug delivery system for the solid tumor. As shown in Supplementary Figure S4, the Sgc8-F-PTX showed better inhibitory effects determined by comparing tumor volumes of the mice from different groups. The results confirm that F-modified ApDCs can deliver the drug to tumor tissue specifically, indicating that F-modified ApDCs are a potential targeted drug-delivery system for cancer therapy.

## 4. Conclusions

In summary, we developed a general approach to elongating the circulatory time for ApDCs. The incorporation of F base into ApDCs was efficiently realized by automated synthesis. In vitro experiments demonstrated that the modification with the F base enhances the stability of aptamers, retaining the binding affinity and targeting the specificity of aptamer-Sgc8. After the injection of the ApDC intravenously into MIA PaCa-2-bearing mice, the F base directed the binding of the ApDC with albumin, and the formulation of the albumin-ApDC complex promoted the ApDC accumulation in the tumor site, while the F-modified ApDC did not exhibit better in vivo inhibitory activity compared with either Nab-PTX or DPBS buffer, which may be caused by the relatively low administration dosage (2 mg/kg) and the large tumor volume (300 mm^3^) at the first-time injection. However, the analysis of PTX concentration in tumor tissue demonstrated that the toxin had been delivered to the tumor specifically and was released efficiently. Our results indicate that F base modification could provide a concise and practical delivery tool for ApDCs with potent applications in targeted cancer therapy.

## Data Availability

The data presented in this work are available in the article and Supplementary Materials.

## Supplementary Materials

The following supporting information can be downloaded at: https://www.mdpi.com/article/10.3390/pharmaceutics14122781/s1, Figure S1: Flow cytometry results of target HCT116 Cells. Cells were respectively incubated with 250 nM Cy5-labeled oligonucleotides at 4 °C for 30 min; Figure S2: In vivo fluorescence imaging of MIA PaCa-2 tumor-bearing mice after Cy5-labeled Sgc8 or Sgc8-FG-PTX were injected intravenously. The tumor sites are marked with yellow circles. (b) Ex vivo imaging of the main organs (heart, liver, spleen, lung, kidney) at 55 h postinjection; Figure S3: Cytotoxicity of MIA PaCa-2 cells after 72 h treatment of a range of concentrations of aptamer derivatives. The concentration range was from 1 to 200 nM. Data were presented as the means ± standard deviation. *n* = 3; Figure S4: The in vivo anti-tumor activity of Sgc8-F-PTX. Analysis of tumor volume of MIA PaCa-2 tumor-bearing mice during different treatments; Table S1: Sequence information of ONs; Table S2: IC50 values of Sgc8-F-PTX, Sgc8-PTX, Nab-PTX, PTX-linker and Sgc8-F after 72h incubation with HCT116, and MIA PaCa-2 cells; Table S3: IC50 values of Sgc8-F-PTX, Sgc8-FG-PTX, PTX, Gemcitabine and PTX together with Gemcitabine after 72h incubation with MIA PaCa-2 cells; Figure S5. 1H-NMR, 13C-NMR, and mass spectra of PTX-linker; Figure S6–S11: The HPLC chromatogram and mass spectra of Sgc8-PTX, Sgc8-F-PTX, Sgc8-F-PTX-cy5, Sgc8-FG-PTX, and Sgc8-FG-PTX-cy5.
