# Supplementary material for: Artificial Base-Directed In Vivo Formulation of Aptamer–Drug Conjugates with Albumin for Long Circulation and Targeted Delivery
Source: Pharmaceutics. 2022 Dec 13;14(12):2781. doi: 10.3390/pharmaceutics14122781 (PMC9781099; doi:10.3390/pharmaceutics14122781)
Supplement: Supplementary file 1 [file pharmaceutics-14-02781-s001.zip › pharmaceutics-2008892-supplementary.pdf]

Supporting Information

Artificial Base-Directed In Vivo Formulation of Aptamer-Drug Conjugates with Albumin for Long Circulation and Targeted Delivery

Yang Sun[1,], Xinyao Geng[1,], Yue Ma[2], Yu Qin[1], Shangjiu Hu[1], Yuquan Xie[2] and Ruowen Wang[1]

[1] Institute of Molecular Medicine (IMM), Renji Hospital, State Key Laboratory of Oncogenes and Related Genes, School of Medicine, Shanghai Jiao Tong University, Shanghai 200127, China

[2] Department of Cardiology, Renji Hospital, State Key Laboratory of Oncogenes and Related Genes, School of Medicine, Shanghai Jiao Tong University, Shanghai 200127, China

Table of Contents

Materials and Methods…………………………………………………………………………………………..3

General Information…………………………………………………………………………………………..3

Cell Culture……………………………………………………………………………………………………3

PTX Precursor Synthesis………………………………………………………………………………………3

Aptamer-PTX Conjugateion……………………………………………………………………………………..3

Specificity and Binding Ability Analysis………………………………………………………………………..4

Figure S1………………………………………………………………………………………………………4

In vivo and *ex vivo* Imaging……………………………………………………………………………………….5

Figure S2……………………………………………………………………………………………………….5

Confocal Microscopy Imaging…………………………………………………………………………………6

Cell Viability Assay……………………………………………………………………………………………6

Figure S3……………………………………………………………………………………………………….6

Serum Stability Analysis………………………………………………………………………………………7

Tumor PTX Content Analysis…………………………………………………………………………………7

Figure S4………………………………………………………………………………………………………8

Supplemental Tables…………...………………………………………………………………….................8

Table S1……………………………………………………………………………………………………….8

Table S2………………………………………………………………………………………………………..9

Table S3………………………………………………………………………………………………………..9

NMR and Mass Spectra Results………………………………………………………………………………..9

Figure S5………………………………………………………………………………………………………11

Figure S6………………………………………………………………………………………………………12

Figure S7……………………………………………………………………………………………………….13

Figure S8……………………………………………………………………………………………………….14

Figure S9……………………………………………………………………………………………………….15

Figure S10……………………………………………………………………………………………………...16

Figure S11……………………………………………………………………………………………………...17

General Information

Unless otherwise noted below, all commercially available reagents and solvents were purchased from Tansoole (Shanghai) and used without further purification. NMR spectra were recorded on a Bruker AM400 spectrometer in our laboratory. All oligonucleotides were synthesized and purified by Sangon Biotech (Shanghai, China) or Biosyn Biotech (Suzhou, China), and the sequences are listed in Table S2. Nab-PTX was purchased from CSPC Pharmaceutical Group Limited. Bovine serum albumin (BSA) and Cell counting kit 8 (CCK-8) were purchased from Beyotime Biotech. All aptamer-drug conjugates were purified by reversed-phase HPLC on a C18 column using 0.1 M triethylamine acetate (TEAA) and acetonitrile as eluent.

Cell Culture

All cells were cultured at 37 °C with 5% CO_2 and 95% humidity. HCT116 cells were maintained in RPMI-1640 medium with 10%FBS (Gibco). MIA PaCa-2 cells were maintained in DMEM medium with 10% FBS. HK-2 cells were maintained in MEM medium with 10% FBS. All cell lines were preserved in our laboratory.

PTX Precursor Synthesis

In the presence of 2,6-dimethylpyridine (28.2 mg, 0.263 mmol), 3-Maleimidopropionic acid (23.4 mg, 0.125 mmol) was added dropwise to a solution of paclitaxel (106 mg, 0.125 mmol) in CH_2Cl_2 (5mL) at 0 ℃. The mixture was stirred at RT for 18 h. When the crude material was consumed, as shown by TLC, the reaction was diluted with CH_2Cl_2 (20 mL) and washed with saturated sodium bicarbonate and saturated saline. After drying over anhydrous sodium sulfate, the solution was concentrated, and the residue was purified by flash column giving PTX precursor as a white powder (75.4 mg, 60% yield).

Aptamer-PTX Conjugation

To the solution of PTX precursor (1200 µL, 2 mM in DMF) was added 1200 µL thiol-modified DNA (200 µM in double distilled H_2O). After ultrasonic vibration for one hour, the mixture was then gently stirred at room temperature for 24 hours. The DNA products were precipitated with NaCl (3 M, 132 µL) and ethanol (6000 µL). Later, the DNA precipitation was diluted to 1400µL with 0.1 M TEAA and purified by HPLC using 0.1 M TEAA and acetonitrile as the eluent with a detection wavelength of 260 nm. The reaction product was finally identified by mass spectrometry.

Specificity and Binding Ability Analysis

The binding affinity of ApDCs was studied by flow cytometry (BD & FACSVerse). The adherent cells were treated with 0.2% EDTA at first, and after three times washing, cells (3×10^5) were incubated with 250 nM Cy5-labeled aptamer derivatives in 200 µL binding buffer on ice for 30 min. Finally, the cells were suspended in 200 µL washing buffer for flow cytometric analysis. Lib was used as a negative control, and Sgc8 was used as a positive control. The Data was analyzed with FlowJo software.

Washing buffer: 5 mM $MgCl_2$ and 4.5 g/L glucose in Dulbecco's PBS.

Binding buffer: 5 mM $MgCl_2$, 4.5 g/L glucose, 0.1 mg/mL yeast tRNA and 1mg/mL BSA in Dulbecco's PBS.

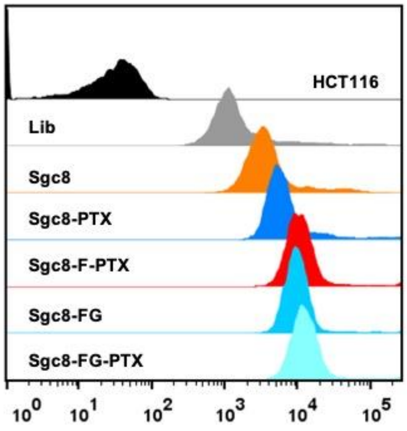

Figure S1. Flow cytometry results of target HCT116 Cells. Cells were respectively incubated with 250 nM Cy5-labeled oligonucleotides at 4 ℃ for 30 min;

In vivo and *ex vivo* Imaging

BALB/c nude mice (4-6 weeks) were provided by the Jihui laboratory animal center (Shanghai, China). All mice were well housed and fed under specific pathogen-free (SPF) conditions. The procedures of all in vivo studies have gained ethical approval from the Institutional Animal Care and Use Committee of Shanghai Jiao Tong University School of Medicine. Mice were euthanized when the tumor size reached more than 2500 mm^3.

Five-week-old female BALB/c nude mice were inoculated subcutaneously with 4×10^6 MIA PaCa-2 cells into the back of the right hind. When the volumes of tumors reached 300-400 mm^3, mice were randomly divided into two groups and intravenously injected with 1 nmol (100 μL of 10 μM) cy5-labeled Sgc8 and Sgc8-FG-PTX respectively. Then the nude mice were anesthetized and imaged with the IVIS Lumina XR imaging system (ex: 640 nm; filter: 670 nm) at predetermined time points including 0, 0.5, 1, 2, 3, 4, 6, 10, 24, 48 and 55 h. The mice were euthanized 55 h after injection. Tumors and major organs (hearts, lungs, livers, spleens, and kidneys) were collected for imaging. Fluorescence intensity was analyzed with GraphPad.

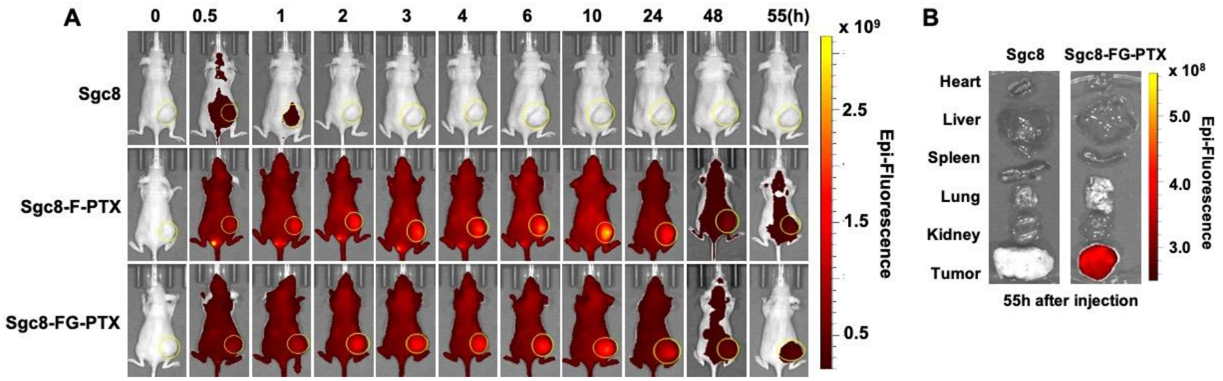

Figure S2. *In vivo* fluorescence imaging of MIA PaCa-2 tumor-bearing mice after Cy5-labeled Sgc8 or Sgc8-FG-PTX were injected intravenously. The tumor sites are marked with yellow circles. (**b**) *Ex vivo* imaging of the main organs (heart, liver, spleen, lung, kidney) at 55 h postinjection.

Confocal Microscopy Imaging

HCT116 and MIA PaCa-2 cells were respectively seeded in glass bottom dishes (NEST Biotechnology) at a density of 1×10^5 per well and allowed to grow overnight. 500 nM Cy5-labeled Sgc8-F-PTX or Cy5-labeled Lib was incubated with cells in a binding buffer at 4 ℃ for 0.5 h. Then, the buffer was removed, and cells were washed three times with washing buffer and replaced with medium supplemented with 10% FBS. After incubating at 37 ℃ for 1 h, the fluorescence of samples was visualized by confocal microscopy (Leica TCS SP8).

Cell Viability Assay

HCT116 and MIA PaCa-2 cells were seeded at a density of 1×10^4 cells or 7×10^3 cells in each well in 96-well plates (Corning) and allowed to grow 24 h for adherence. A range of concentrations from 1 to 200 nM of samples were made up in medium and added into cells for 72 h incubation at 37 °C. Cell viability was assayed using CCK-8 kit by monitoring the absorbance at 450 nm with a microplate reader (Biotek, Synergy H1). Finally, IC50 values of drugs were calculated using GraphPad based on the viability curve data. Data were mean ± SD, n=3.

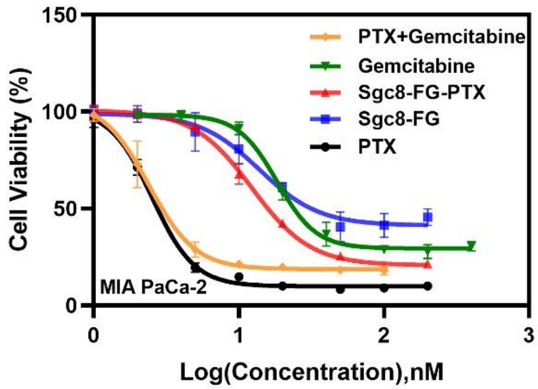

Figure S3. Cytotoxicity of MIA PaCa-2 cells after 72 h treatment of a range of concentrations of aptamer derivatives. The concentration range was from 1 to 200 nM. Data were presented as the means ± standard deviation. n = 3.

Serum Stability Analysis

To investigate the serum stability of aptamers, 1 μM Cy5-labeled Sgc8 and Sgc8-F-PTX were incubated with DMEM with 10% fetal bovine serum (FBS) at 37 °C. At predetermined time points including 0, 1, 3, 6, 8, 12, 24, 48 and 72 h, samples were taken out and heated at 95 °C for 5 min and subsequently stored at -20 °C. When all samples were collected, the samples mixed with DNA loading buffer were analyzed by 10% polyacrylamide gel electrophoresis (PAGE) at 100 V for 40 min. Finally, the gels were imaged using the GE & Amersham Imager 680R.

Tumor PTX Content Analysis

When the volume of tumors reached about 300 mm^3, mice were randomly divided into four groups including Dulbecco's Phosphate Buffered Saline (DPBS), Sgc8-F-PTX, Nab-PTX and Sgc8-F. Sgc8-F-PTX, Nab-PTX, Sgc8-F, at a dosage of 2.34 μmol/kg (with the equivalent PTX concentration of 2 mg/kg) were administered to mice by intravenous tail injection once a week for 2 weeks. After 2 weeks' therapy, the DNA dose was increased to 4.68 μmol/kg (equal to a dose of 4 mg/kg for PTX) during the next 2 weeks. After administration, the mice were observed for

another 2 weeks. The vehicle solution DPBS was used as the control group. The tumor size and body weight were monitored twice a week. At the end of the treatment, mice were sacrificed, tumors and major organs (hearts, lungs, livers, kidneys, and spleens) were collected and weighed. After 6 weeks' therapy, the tumors of all treatment groups were collected and weighed. The content of PTX in tumors was carried out by Sangon Biotech using UPLC-ESI-MS/MS. Drug content was analyzed with GraphPad.

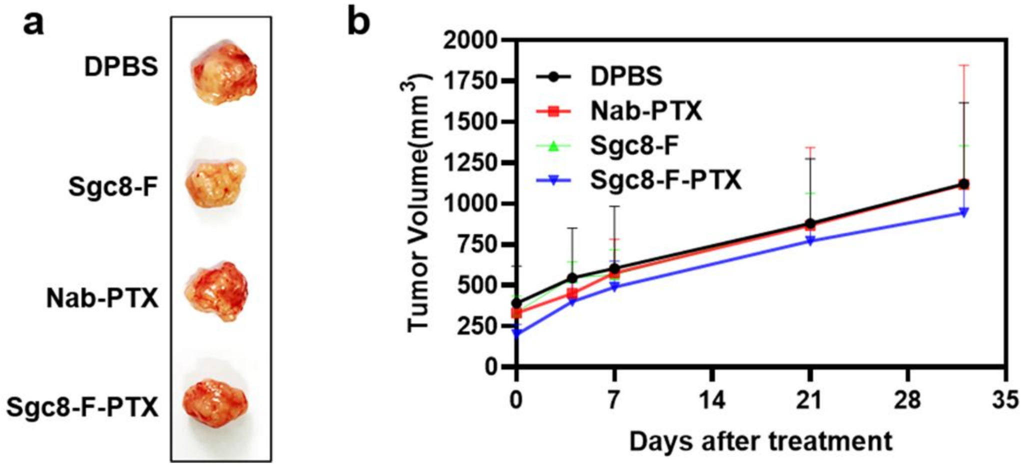

Figure S4. The *in vivo* anti-tumor activity of Sgc8-F-PTX. Analysis of tumor volume of MIA PaCa-2 tumor-bearing mice during different treatments.

Supplemental Tables

Table S1 Sequence information of ONs

ON	Sequence (5' to 3')
Sgc8	ATCTAACTGCTGCGCCGCCGGGAAAATACTGTACGGTTAGA
Sgc8-F	FFATCTAACTGCTGCGCCGCCGGGAAAATACTGTACGGTTAGAFF

Sgc8-F-G	FFATCTAACTGCTGCGCCGCCGGGADAATA DTGTACGGTTAGAFF
C_6-SH-Sgc8	SH-C_6-ATCTAACTGCTGCGCCGCCGGGAAAATACTGTACGGTTAGA
C_6-SH-Sgc8-F	SH-C_6-FFATCTAACTGCTGCGCCGCCGGGAAAATACTGTACGGTTAGAFF
C6-SH-Sgc8-F-G	SH-C6-FFATCTAACTGCTGCGCCGCCGGGADAATADTGTACGGTTAGAFF
Sgc8-Cy5	ATCTAACTGCTGCGCCGCCGGGAAAATACTGTACGGTTAGA-Cy5
C_6-SH-Sgc8 -Cy5	SH-C_6-ATCTAACTGCTGCGCCGCCGGGAAAATACTGTACGGTTAGA-Cy5
C_6-SH-Sgc8-F-Cy5	SH-C_6-FFATCTAACTGCTGCGCCGCCGGGAAAATACTGTACGGTTAGAFF-Cy5
C6-SH-Sgc8-F-G-Cy5	SH-C6-FFATCTAACTGCTGCGCCGCCGGGADAATADTGTACGGTTAGAFF-Cy5
Lib-Cy5	NNNNNNNNNNNNNNNNNNNNNNNNNNNNNNNNNNNNNNNNN-Cy5

F represents the artificial base from phosphoramidite F, and D represents a Gemcitabin unit from commercially available D phosphoramidite.

Table S2 IC50 values of Sgc8-F-PTX, Sgc8-PTX, Nab-PTX, PTX-linker and Sgc8-F after 72h incubation with HCT116, and MIA PaCa-2 cells.

Cell line	IC50(nM)				
	Sgc8-F	PTX-linker	Nab-PTX	Sgc8-PTX	Sgc8-F-PTX
HCT116	N/A	12.64	7.816	51.81	13.83
MIA PaCa-2	N/A	4.235	11.58	44.72	15.04

Table S3 IC50 values of Sgc8-F-PTX, Sgc8-FG-PTX, PTX, Gemcitabine and PTX together with Gemcitabine after 72h incubation with MIA PaCa-2 cells.

Cell line	IC50(nM)				
	PTX	Gemcitabine	PTX with Gemcitabine	Sgc8-F-PTX	Sgc8-FG-PTX
MIA PaCa-2	1.93	20.75	2.90	18.98	13.24

NMR and Mass Spectra Results

a

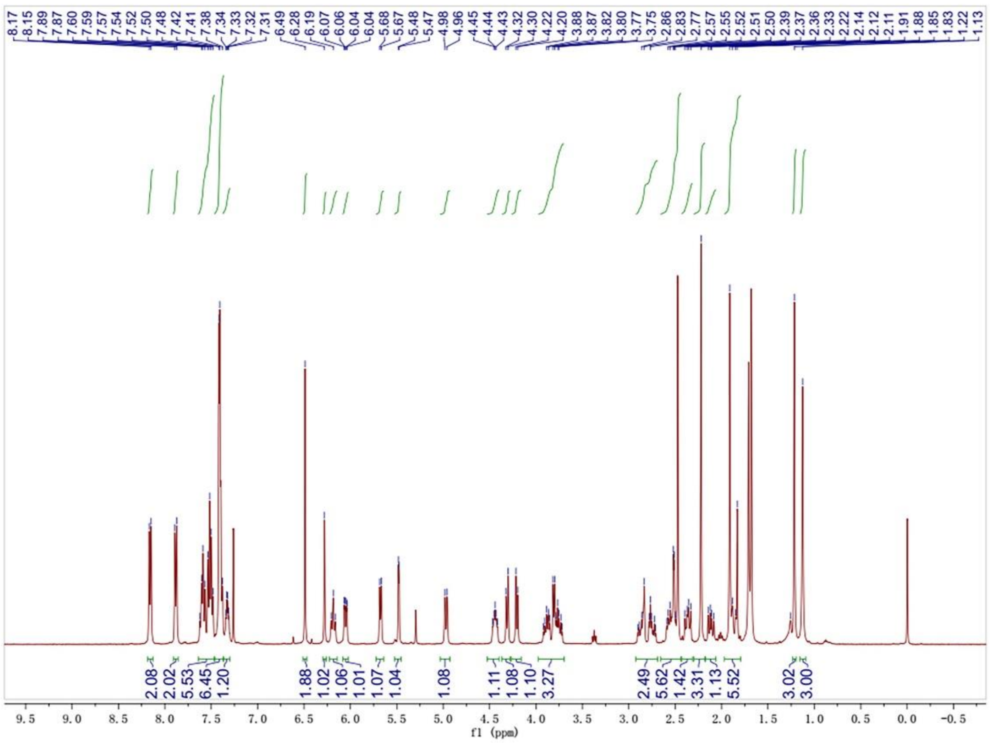

b

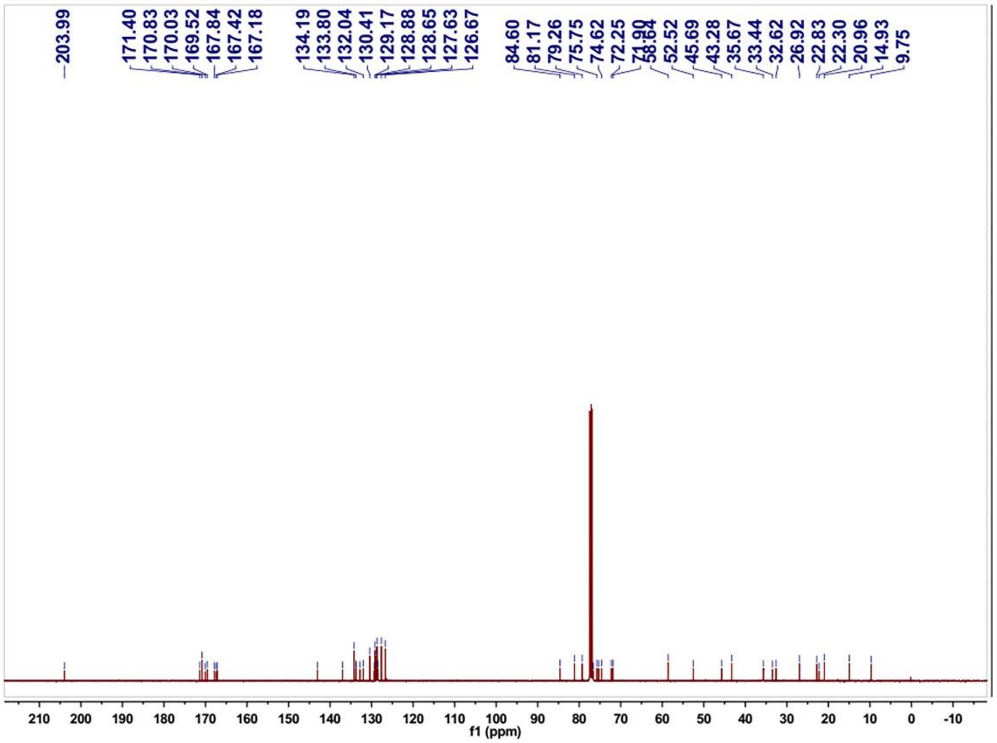

c

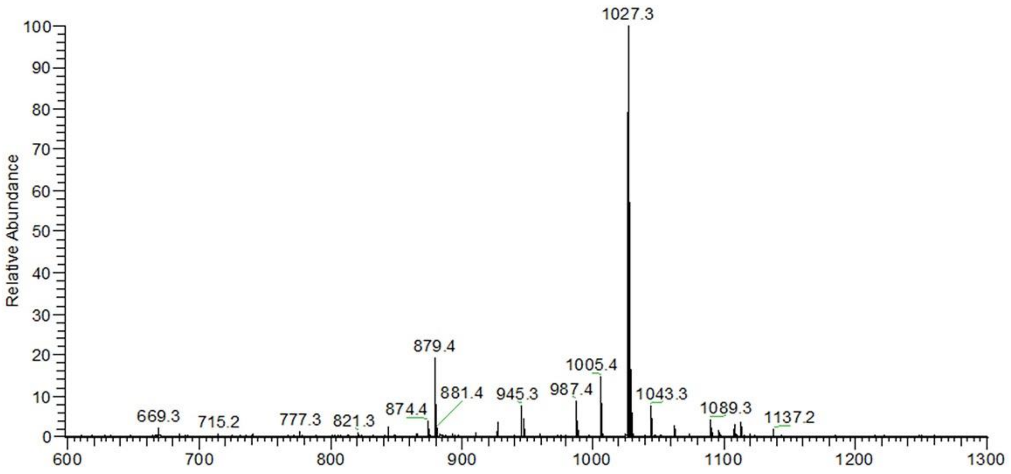

Figure S5. ^{1}H-NMR, ^{13}C-NMR, and mass spectra of PTX-linker. (a) The ^{1}H-NMR spectrum of PTX-linker (400 MHz, $CDCl_3$), δ 8.16 (d, 2H), 7.88 (d, 2 H), 7.62-7.48 (m, 5 H), 7.41 (m, 6 H), 7.34-7.31 (m, 1 H), 6.49 (s, 2 H), 6.28 (s, 1 H), 6.19 (t, 1 H), 6.05 (dd, 1 H), 5.68 (d, 1 H), 5.48 (d, 1 H), 4.97 (d, 1 H), 4.49-4.40 (m, 1 H), 4.31 (d, 1 H), 4.21 (d, 1 H), 3.92-3.72 (m, 3 H), 2.95-2.69

(m, 2 H), 2.64-2.44 (m, 5 H), 2.36 (m, 1 H), 2.22 (s, 3 H), 2.11 (m, 1 H), 1.96-1.81 (m, 5 H), 1.24 (s, 3 H), 1.13 (s, 3 H); (**b**) The ^{13}C-NMR spectrum of PTX-linker (100 MHz, $CDCl_3$), δ 203.99, 171.40, 170.83, 170.03, 169.52, 167.84, 167.42, 167.18, 143.00, 137.01, 134.19, 133.80, 133.71, 132.82, 132.04, 130.41, 129.36, 129.17, 128.88, 128.65, 128.43, 127.63, 126.67, 84.60, 81.17, 79.26, 76.58, 75.75, 75.26, 74.62, 72.25, 71.90, 58.64, 52.52, 45.69, 43.28, 35.67, 33.44, 32.62, 26.92, 22.83, 22.30, 20.96, 14.93, 9.75;(**c**) The mass spectrum of PTX-linker, MS (ESI):$[M+Na]^+$: calculated 1027.3, found 1027.3, HRMS calculated for $[C54H56N2O17\ Na]^+$ 1027.3471, found 1027.3489.

a

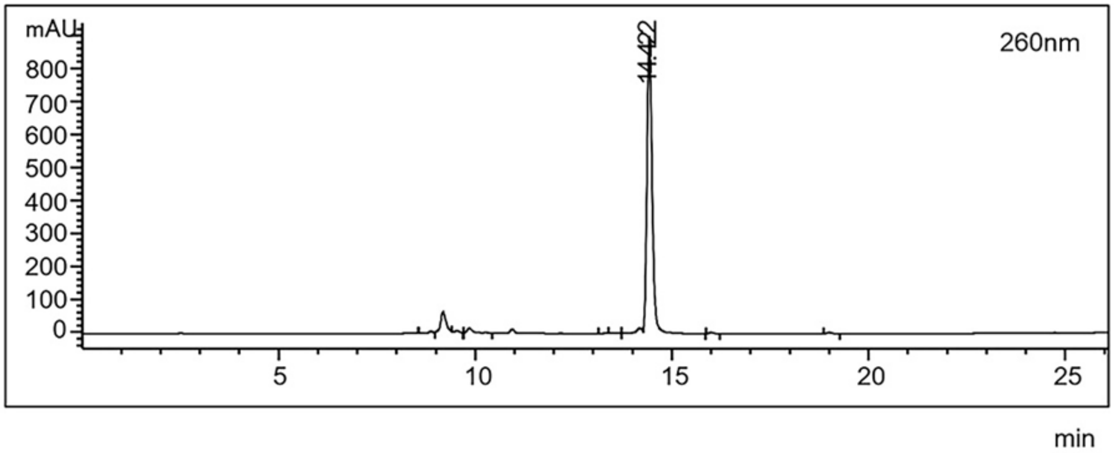

b

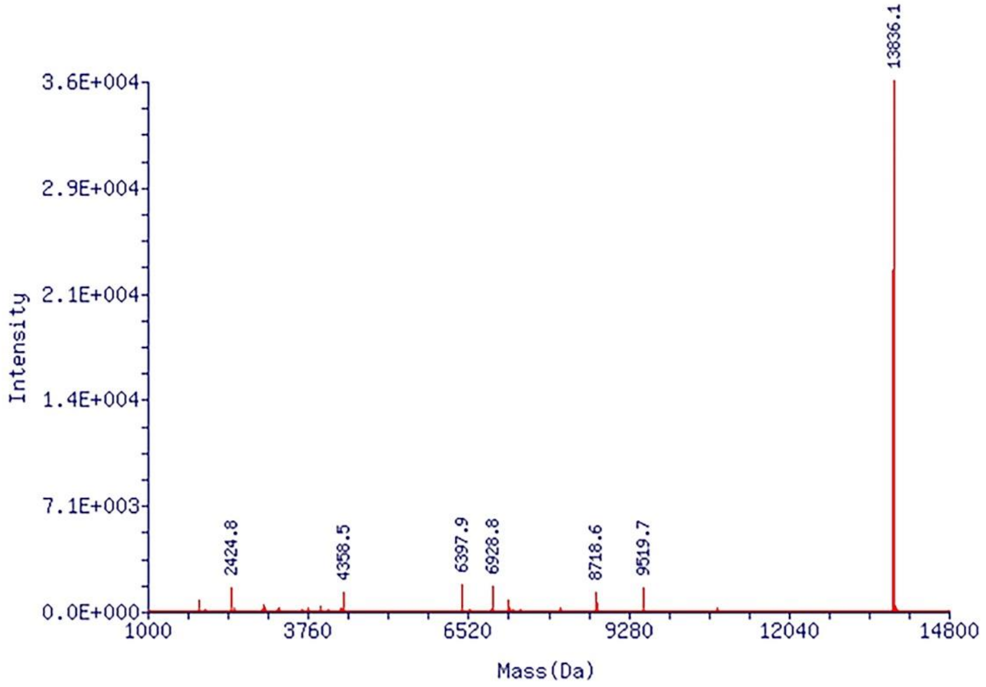

Figure S6. The HPLC chromatogram and mass spectra of Sgc8-PTX. (a) The HPLC chromatogram of the Sgc8-PTX. (**b**) Mass analysis of Sgc8-PTX by Sangon (Shanghai). Calculated molecular weight:13835.3, Found: 13836.1.

a

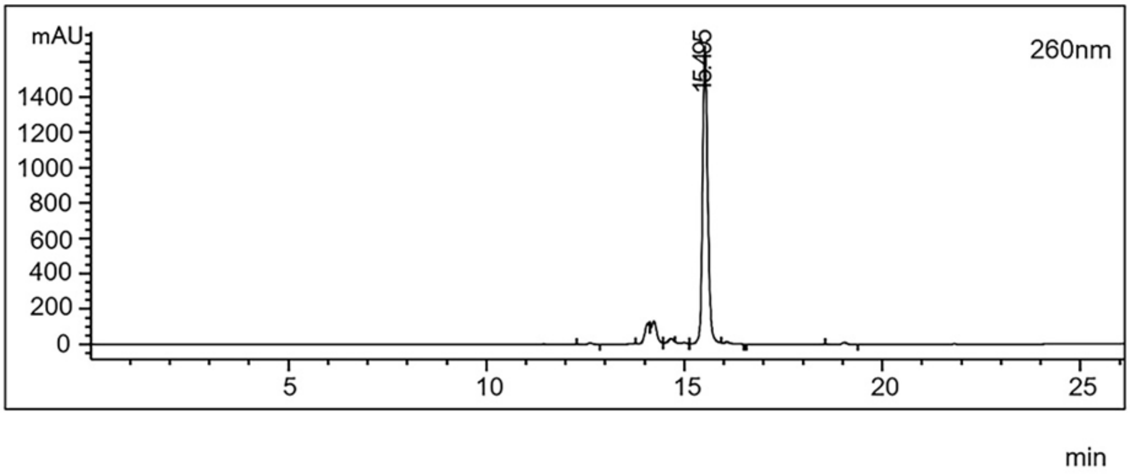

b

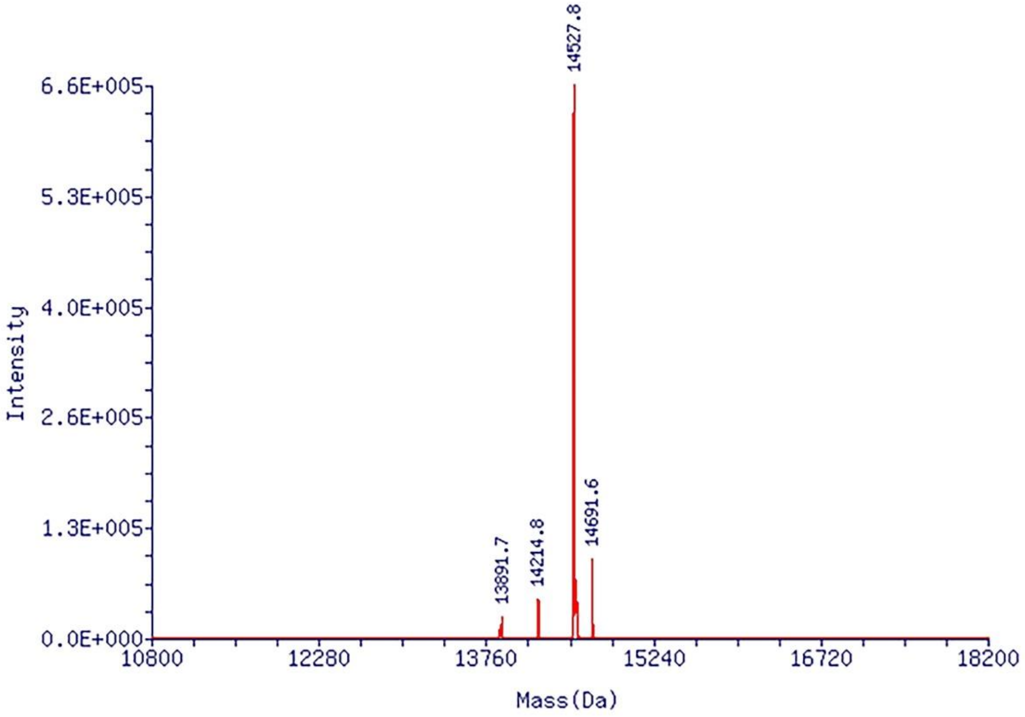

Figure S7. The HPLC chromatogram and mass spectra of Sgc8-PTX-cy5. (a) The HPLC chromatogram of the Sgc8-PTX-cy5. (**b**) Mass analysis of Sgc8-PTX-cy5 by Sangon (Shanghai). Calculated molecular weight :14524.1, Found: 14527.8.

a

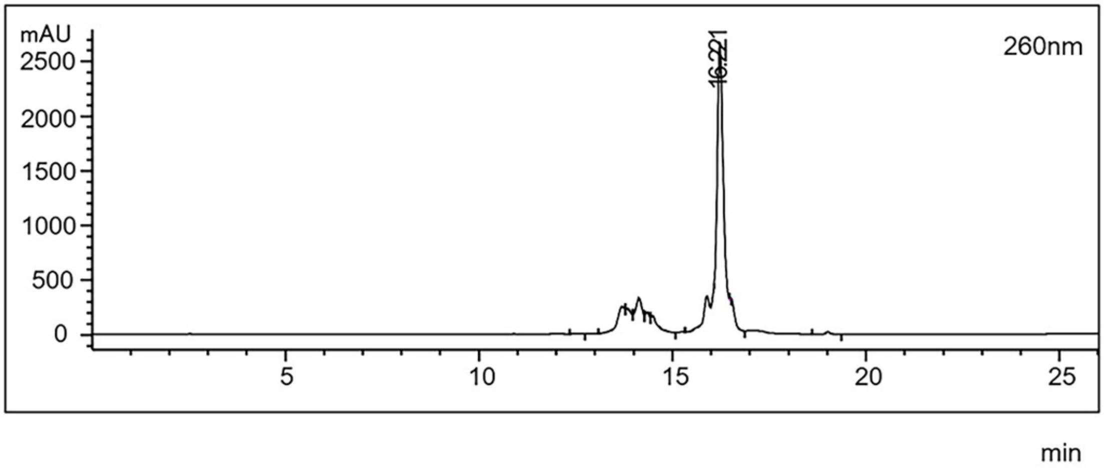

b

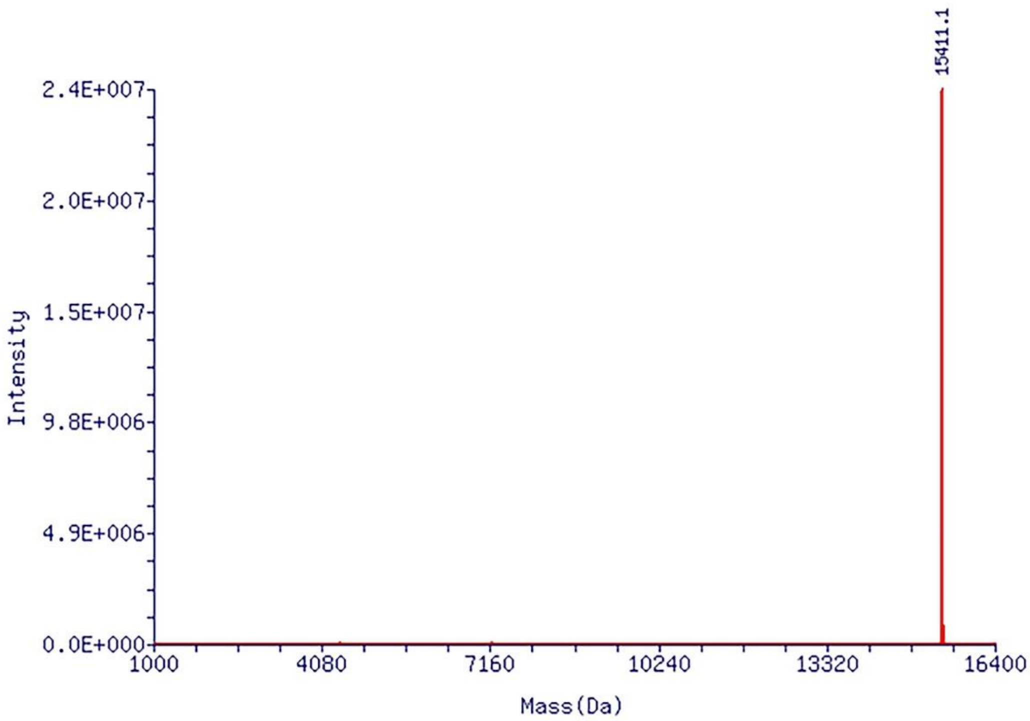

Figure S8. The HPLC chromatogram and mass spectra of Sgc8-F-PTX. (a) The HPLC chromatogram of the Sgc8-F-PTX. (**b**) Mass analysis of Sgc8-F-PTX by Sangon (Shanghai). Calculated molecular weight :15407.2, Found: 15411.1.

a

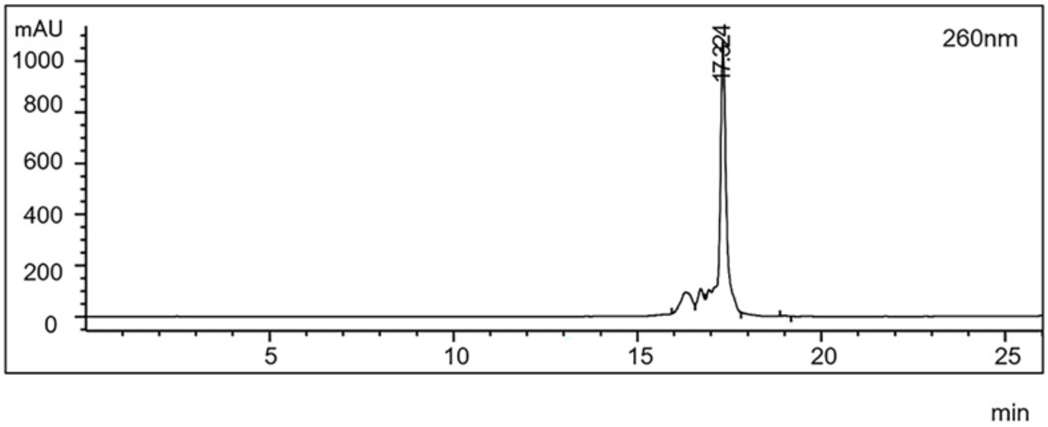

b

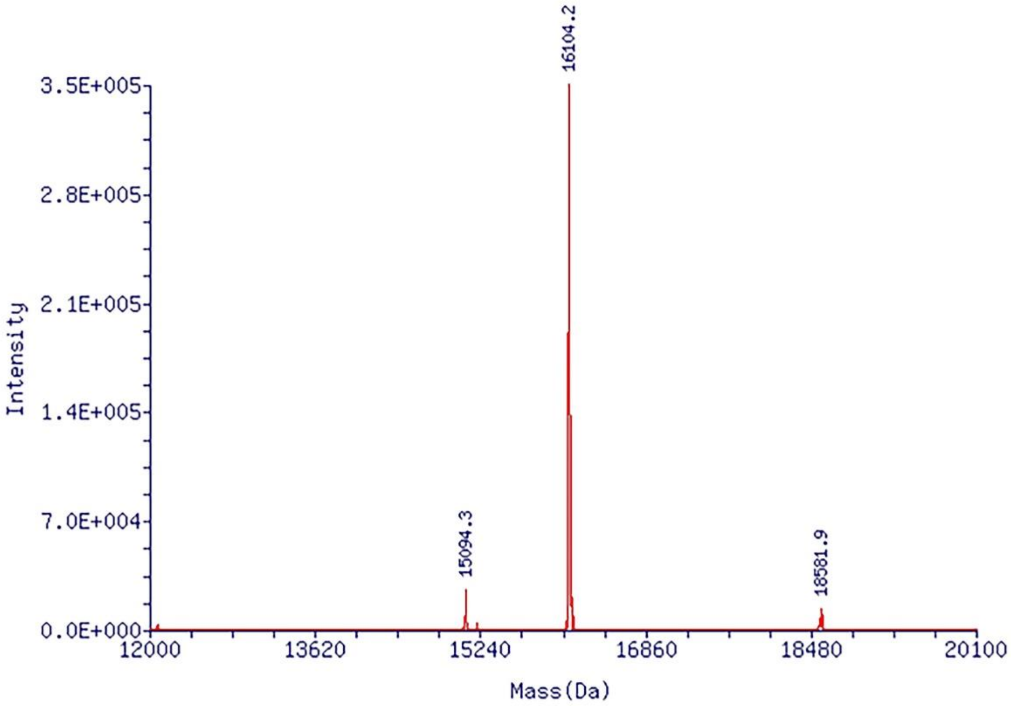

Figure S9. The HPLC chromatogram and mass spectra of Sgc8-F-PTX-cy5. (a) The HPLC chromatogram of the Sgc8-F-PTX-cy5. (**b**) Mass analysis of Sgc8-F-PTX-cy5 by Sangon (Shanghai). Calculated molecular weight:16095.2, Found: 16104.2.

a

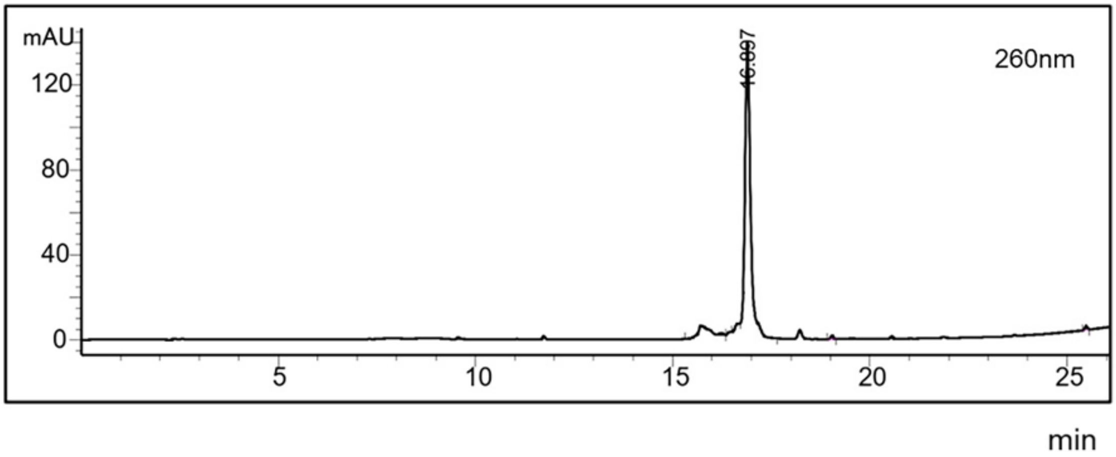

b

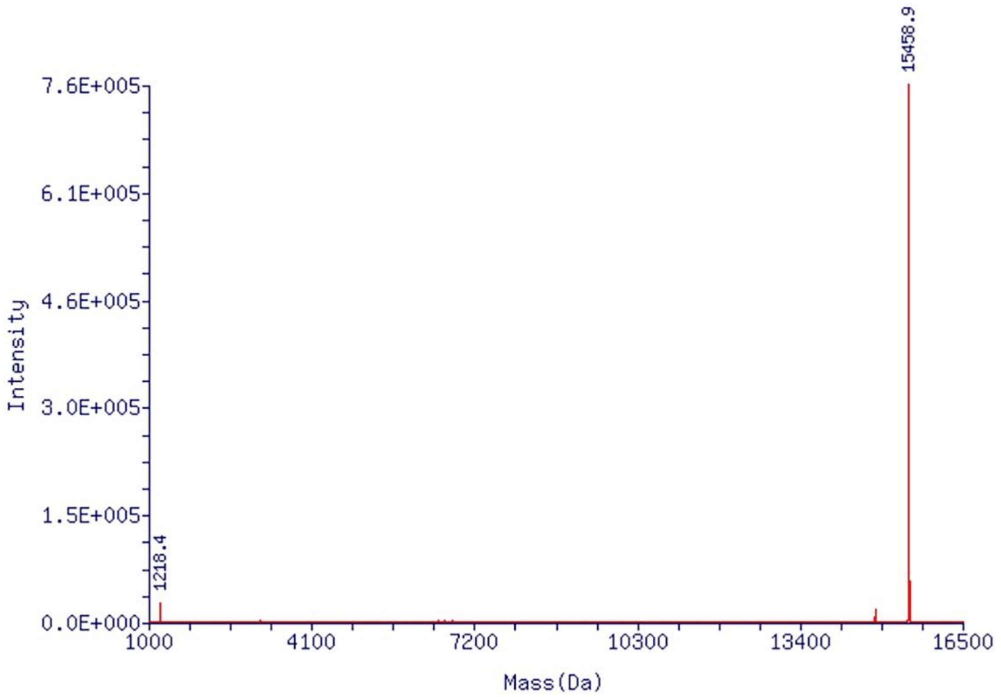

Figure S10. The HPLC chromatogram and mass spectra of Sgc8-FG-PTX. (a) The HPLC chromatogram of the Sgc8-FG-PTX. (**b**) Mass analysis of Sgc8-FG-PTX by Sangon (Shanghai). Calculated molecular weight:15455.2, Found: 15458.9.

a

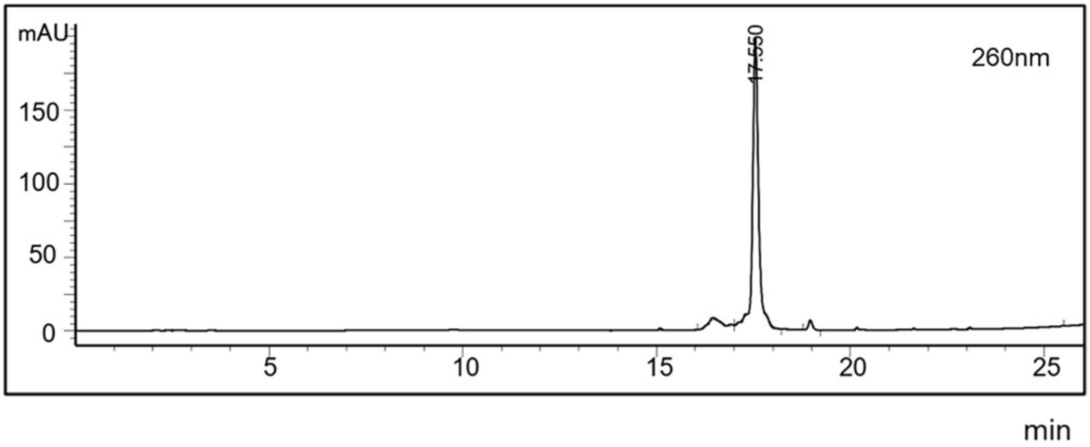

b

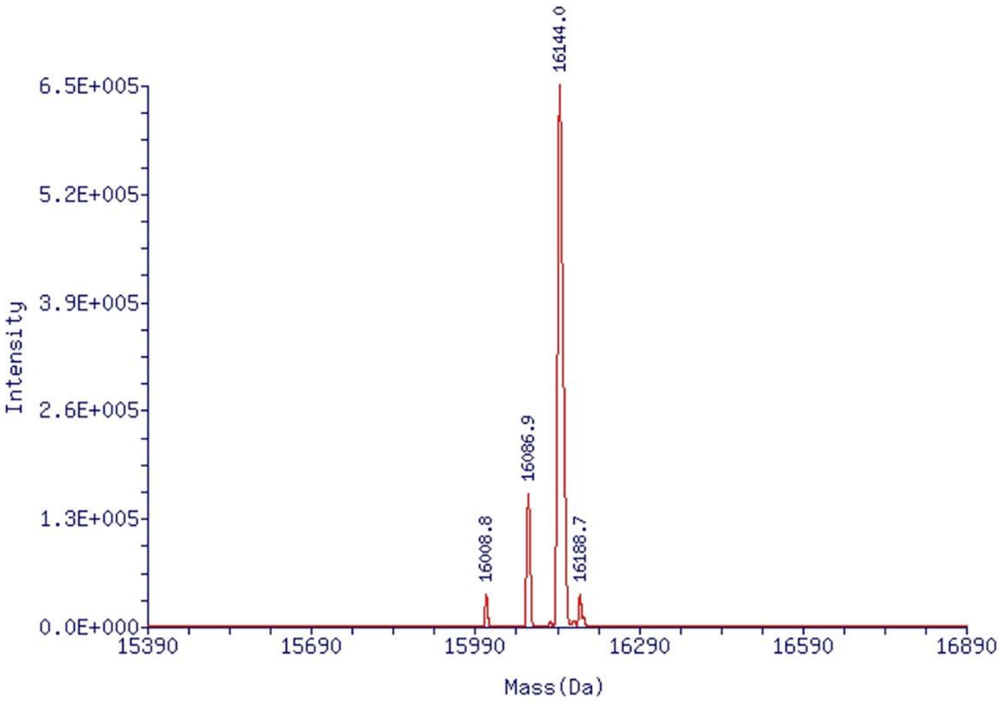

Figure S11. The HPLC chromatogram and mass spectra of Sgc8-FG-PTX-cy5. (a) The HPLC chromatogram of the Sgc8-FG-PTX-cy5. (**b**) Mass analysis of Sgc8-FG-PTX-cy5 by Sangon (Shanghai). Calculated molecular weight:16143.2, Found: 16144.
